# Parental exposure to elevated pCO_2_ influences the reproductive success of copepods

**DOI:** 10.1093/plankt/fbu052

**Published:** 2014-06-19

**Authors:** Gemma Cripps, Penelope Lindeque, Kevin Flynn

**Affiliations:** 1 CSAR, Swansea University, Swansea SA2 8PP, UK; 2 Plymouth Marine Laboratory, Prospect Place, West Hoe, Plymouth PL1 3DH, UK

**Keywords:** elevated pCO_2_, copepods, parental exposure, egg hatching rates, spermatogenesis, ocean acidification

## Abstract

Substantial variations are reported for egg production and hatching rates of copepods exposed to elevated carbon dioxide concentrations (pCO_2_). One possible explanation, as found in other marine taxa, is that prior parental exposure to elevated pCO_2_ (and/or decreased pH) affects reproductive performance. Previous studies have adopted two distinct approaches, either (1) expose male and female copepoda to the test pCO_2_/pH scenarios, or (2) solely expose egg-laying females to the tests. Although the former approach is more realistic, the majority of studies have used the latter approach. Here, we investigated the variation in egg production and hatching success of *Acartia tonsa* between these two experimental designs, across five different pCO_2_ concentrations (385–6000 µatm pCO_2_). In addition, to determine the effect of pCO_2_ on the hatching success with no prior parental exposure, eggs produced and fertilized under ambient conditions were also exposed to these pCO_2_ scenarios. Significant variations were found between experimental designs, with approach (1) resulting in higher impacts; here >20% difference was seen in hatching success between experiments at 1000 µatm pCO_2_ scenarios (2100 year scenario), and >85% at 6000 µatm pCO_2_. This study highlights the potential to misrepresent the reproductive response of a species to elevated pCO_2_ dependent on parental exposure.

## INTRODUCTION

Mesozooplankton play a pivotal role in marine food webs, mediating the transfer of primary production to higher trophic levels. Copepods are the most abundant organisms of the mesozooplankton and in consequence any potential effect on their productivity and population structure, as a result of ocean acidification (OA), will likely impact on marine ecology and biogeochemical cycling. Sub-lethal responses to increased concentrations of carbon dioxide (pCO_2_) have been shown to vary considerably between copepod species, particularly with regards to reproductive success. While reduced egg production, hatching rates and naupliar production have been found in some species (e.g. *Acartia tonsa, Tisbe battagliai*) exposed to pCO_2_ lower than that projected for 2100 year scenario [i.e. ≤1000 µatm pCO_2_, (Fitzer *et al.*, 2012b, 2013; Rossoll *et al.*, 2012)], other species (e.g. *Centropages typicus, C. finmarchicus* and *Temora longicornis*) have shown no reproductive effects upon exposure to concentrations that are more than twenty times the current level [8–10 000 µatm CO_2_, (Mayor *et al.*, 2007; McConville *et al.*, 2013)].

This variation in reproductive response could in part be attributed to experimental design, particularly with respect to which parents (i.e. males, females or both) have been exposed to the elevated pCO_2_. Previous OA studies have adopted two different experimental approaches to measure reproductive success (i.e. egg production and hatching rates) in copepods: (1) exposure of both males and females to the pCO_2_/pH scenario, (2) sole exposure of egg-laying females to the pCO_2_/pH scenario. The majority of studies have utilized the latter approach, whereby the reproductive output is influenced solely through maternal exposure to elevated pCO_2_. Within these studies, there have been no reproductive effects found under the 2100 year scenario (≤1000 µatm pCO_2_), with impacts only being found at concentrations that far exceed any climate change projection. In stark contrast, the few studies which have exposed both sexes have revealed the potential for deleterious effects of combined parental exposure to pCO_2_ concentrations as low as 450 µatm (Fitzer *et al.*, 2012b).

**Fig. 1.**
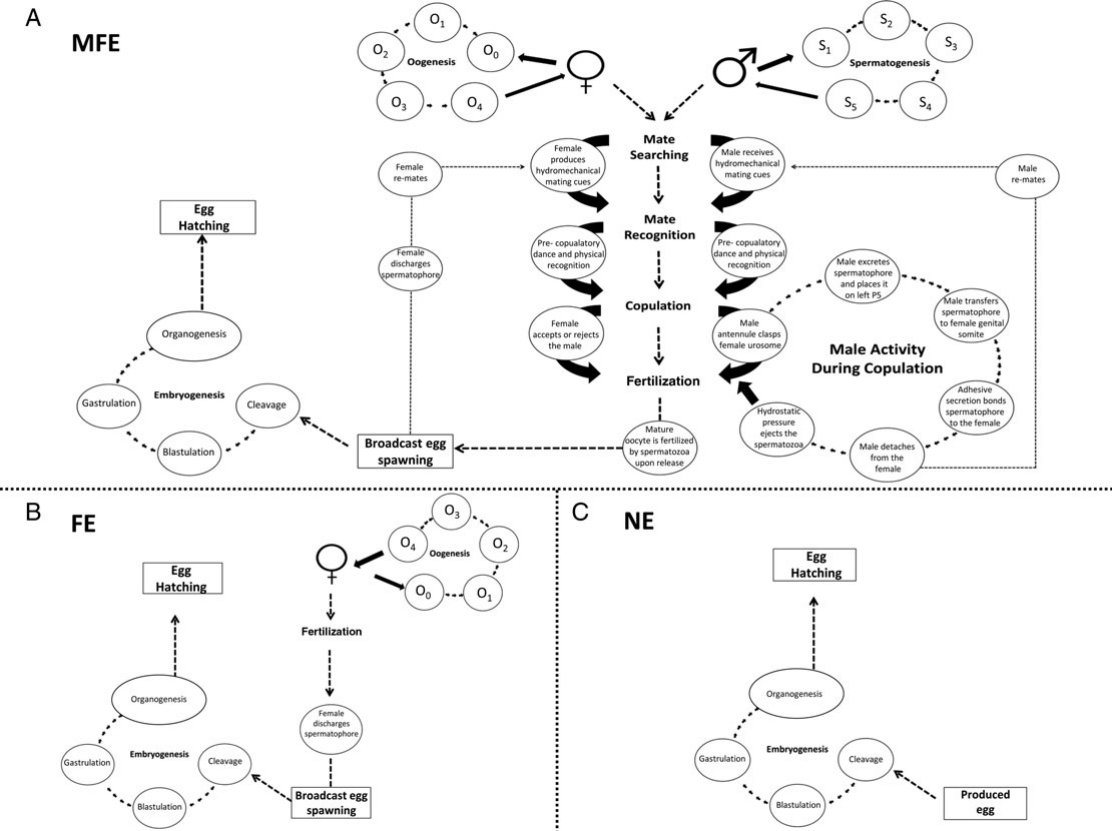
A schematic of the mating, reproduction and embryonic developmental stages of a calanoid broad cast spawner, specifically for *Acartia tonsa,* with differing parental exposures. (**A**) Illustrates the mating, reproduction and embryonic developmental stages during combined MFE. (**B**) Illustrates the reproductive stages and embryonic developmental stages exposed to the pCO_2_ scenario during sole FE. (**C**) Illustrates the egg developmental processes after fertilization that are exposed to the elevated pCO_2_ scenario with no parental exposure (NE). During oogenesis; *Oocyte 0:* the oogonia and previtellogenic oocytes in early meiotic stages. *Oocyte 1*: nucleoli and yolk vesicles present in the oocyte. During this phase, the vitellogenesis is in phase 1. *Oocyte 2*: growth of ooplasm occurs with yolk droplets in the oocyte. *Oocyte 3*: nuclear membrane and nucleolus disintegrate in the oocyte, and condensation of chromosomes occurs. During this phase, the vitellogenesis is in phase 2. *Oocyte 4*: the ooplasm is filled with yolk and lipid vesicles, the nuclear membrane and nucleoli dissolve and the mature oocytes are released during this stage. During spermatogenesis; *S1*: immature spermatozoa produced in the testis passes through the genital duct. *S2*: in the vas deferens the seminal fluid that surrounds the spermatozoa is produced, as well as material for the spermatophore wall. *S3*: additional material for the spermatophore wall is produced as the spermatozoa pass through seminal vesicle. *S4*: within the spermatophore sac, the anterior gland produces adhesive secretions for the spermatophore transfer. The spermatophore sac is then released into the lumen. *S5*: the spermatophore is released from the posterior left side of the genital somite to be transferred to the female.

Exposing both adult males and females to the test pCO_2_ concentration enables *in situ* copulation, fertilization and production under those conditions, so that the reproductive output is influenced by both maternal and paternal exposure to the pCO_2_; thus mimicking events in nature. Figure 1 is a schematic showing the reproductive stages of a calanoid broadcast spawner, specifically *Acartia* sp. and how these are exposed to environmental conditions under different experimental designs. The reproductive, developmental and mating stages that are exposed to the pCO_2_ level mimicking events in nature (i.e. the whole reproductive cycle) are highlighted in Fig. 1A. In contrast, as shown in Fig. 1B, the sole exposure of egg-laying females discounts any effects that the increased pCO_2_ concentration may have on the male gametes and/or to the mating process itself. Instead, sole exposure of egg-laying females only accounts for the effects of high pCO_2_ on any pre-attached spermatophore, stored seminal fluids (assuming that experiments are conducted with females containing sufficient stores of spermatozoa to enable offspring production) and fertilization of the eggs upon release. Thus, the type of experimental design which is most frequently used (Fig. 1B) cannot represent the “true” effects of high pCO_2_ on reproduction; it only represents how maternal exposure to increased levels of pCO_2_ influences egg production and hatching success.

The maternal influence on offspring survival and fitness can be a significant factor in population dynamics across many marine taxa (Fowler, 2005), including copepods (Kahan and Berman, 1988). Maternal awareness of the environmental conditions and cues (food quality and quantity, temperature, competition, population density) may result in changes in investment in reproduction, affecting offspring survivorship [the so-called anticipatory maternal effects, AMEs (Marshall *et al.,* 2008)], and an alteration in larvae/offspring size to suit environmental changes (Pond *et al.*, 1996; Halsband and Hirche, 2001; Parker *et al.*, 2012; Fitzer *et al*., 2013). Equally, mothers may invest differently in reproduction if the environmental conditions are not favourable, resulting in decreased offspring fitness with self-regarding maternal motives [the so-called selfish maternal effects, SMEs (Marshall *et al.,* 2008)]. In studies that solely expose egg-laying females to increased levels of pCO_2_ (Fig. 1B), there is thus a distinct possibility that what is seen are short-term maternal responses to the pre-zygotic pre-/post-natal (species dependent) egg as a result of a rapid changing environment. Without inclusion of the additional paternal influence on the generation of offspring, a skewed understanding of the reproductive effects of OA in copepod populations is possible. Understanding this potential would aid in explaining the current variation in reproductive response of copepods exposed to high pCO_2_ between the two different experimental designs published to date.

The aim of this study, therefore, was to examine the direct variation in reproductive success between these two experimental designs. We separately exposed (i) egg-laying females (as shown in Fig. 1B), and (ii) combined males and females (as shown in Fig. 1A) to five different pCO_2_ levels (385–6000 µatm pCO_2_) and compared their fecundity success through egg production, egg size, hatching rates and nauplii production (NP). Furthermore, to highlight the potential influence of parental exposure to pCO_2_ of the two above experimental designs, we introduced another experimental design as a positive control; (iii) exposing eggs, which had previously been produced and fertilized under ambient conditions (and thus had no prior parental exposure to elevated pCO_2,_ as shown in Fig. 1C), to the five pCO_2_ levels and measured their hatching success.

## METHOD

### Copepods

The calanoid copepod, *Acartia tonsa*, was obtained originally from Environment & Resource Technology (ERT), Orkney, UK. Stock populations were cultured in the Centre of Sustainable Aquatic Research (CSAR), Swansea, UK. Stock cultures were maintained at 24.4°C (± 0.54) with a 14:10 photoperiod (4–9 µmol photons m^−2^ s^−1^) in aerated (392 ± 27 ppm pCO_2_) filtered (0.22 µm) seawater. These stock *Acartia tonsa* were fed *ad libitum* on a mixed microalgae diet of *Isochrysis galbana* (Strain CCAP 927/1), *Tetraselmis suecica* (Strain CCAP 66/22C) and *Chaetoceros muelleri* (Strain CCAP 1010/3). The microalgae were grown separately in a seawater-based *f*/2 medium (Guillard and Ryther, 1962), maintaining a nutrient-replete status [average (±1SD) mass C:N ratios of *Isochrysis* 5.74 ± 0.41, *Tetraselmis* 7.27 ± 0.84 and *Chaetoceros* 6.22 ± 0.50], and were fed to copepods in a ratio of 1:1:1 relative to the carbon biomass concentration of the algae (respectively, the initial cell densities at the time of addition to the copepods were 50 × 10^3^, 4.0 × 10^3^, 25 × 10^3^ cells mL^−1^; total C-biomass added = 1 µg C mL^−1^). Copepods were reared under these conditions until sufficient numbers were obtained for each experimental design.

### Treatment levels

*Acartia tonsa* were exposed to five different pCO_2_ levels: (i) present-day pCO_2_, 385 µatm, (ii) near future level, 1000 µatm (RCP8.5, 2100 year pCO_2_ projection, Vuuren *et al*., 2011), (iii) 2000 µatm (ECP8.5, 2300 year pCO_2_ projection, Vuuren *et al*., 2011) and two extreme pCO_2_ levels (iv) 3000 µatm, and (v) 6000 µatm. The two latter levels were used to determine lethal and sub-lethal threshold limits, both of which correlate to potential carbon capture and storage leakage scenarios (Blackford *et al*., 2009). These different levels of seawater pCO_2_ were obtained through mixing water of a known high pCO_2_ with water saturated with ambient CO_2_, to attain the desired level (Riebesell *et al*., 2010). Measurements of pH were made through a 3-point decimal place Omega PHB-121 bench top microprocessor pH meter cross referenced with a WTW 315i portable meter (2A10–101T), both calibrated with pH 7.01 and 10.01 (NBS scale). Total alkalinity (measured by open cell potentiometric titration using an AS-ALK2 Gran Titrator, Apollo SciTech), pH, salinity and temperature were used to calculate the pCO_2_ (µatm) through the programme CO2SYS (Pierrot *et al*., 2006), using the K1, K2 constants from Mehrbrach *et al.* (Mehrbrach *et al.,* 1973), as refitted by Dickson and Millero (Dickson and Millero, 1987).

### Experimental design

Experimental design and protocols used were similar to that used in Cripps *et al.* (Cripps *et al.,* 2014). Three separate experiments were carried out to determine the variation in reproductive success between the two contrasting experimental approaches: (i) combined male and female exposure (MFE), (ii) sole female exposure (FE), in addition to (iii) a positive control with no parental exposure (NE).

### Male and female exposure (MFE)

Young mature males and females (<1.25-day maturity, virgin females without attached spermatophore) were incubated separately under the five different pCO_2_ treatments for 72 h. For each pCO_2_ level, there were three replicate culture flasks for the males and six replicate culture flasks for the females (volume: 260 mL, concentration of 0.046 individual's mL^−1^ for both). Flasks were rotated on a plankton wheel at 2 rpm in a constant temperature room (24°C), with a 14:10 photoperiod (4–9 µmol photons m^−2^ s^−1^). Water was exchanged every 24 h and prey concentrations were renewed to known saturating conditions (≥1 µg C mL^−1^ of equal carbon concentrations of *I. galbana*, *T. suecica* and *C. mulleri*). After 72 h exposure, males and females within the same pCO_2_ treatment level were combined in a 260 mL tissue culture flask (nine females and three males in each flask with 0.046 ind^−1^ mL^−1^, four replicates per treatment). After 30 h, 10–15 females with an attached spermatophore were selected for egg production rates (EPR) across all treatments. Females were placed individually into a 30 mL universal vial with their assigned pCO_2_ treatment and saturating prey concentrations. Each universal vial was lined with a 150 µm nylon mesh false bottom to prevent female egg cannibalism. The vials were sealed to prevent gaseous exchange altering the pCO_2_ concentrations. After 24 h, EPR were determined for each female across the five pCO_2_ treatments. Subsequently, eggs were used for hatching success (EHS) and size measurements. For EHS, eggs were placed individually into each well (well volume: 3.6 mL) of a 24-well culture plate with the designated pCO_2_ treatment (minimum of three replicate plates per treatment). Hatching success was measured every 24 h for a 96 h period. The number of eggs produced which hatched into nauplii was used to determine the NP per female. The diameter of at least 20 eggs from each pCO_2_ level was measured from digital images (Leica LAS 3.8.0). Eggs were assumed to be spherical, volume being calculated as (4/3)π*r*^3^.

#### Sole female exposure (FE)

Females of mixed maturity (1–3 days) were collected from stock cultures. Mixed ages were chosen to mimic the studies which utilize this method. (The majority of published studies have used copepods caught from the wild, and hence the age of the females is unknown. Indeed, the amount of stored seminal fluids available for reproduction within the individual female is also unknown). For each pCO_2_ treatment, there were four replicate 260 mL culture flasks (0.046 ind^−1^ mL^−1^), which were maintained under the same controlled conditions as described in above for 96 h. The EPR, EHS, NP and egg volume were determined as described above.

#### No parental exposure (NE)

Approximately 3000 females of mixed maturity (1–5 days) were divided between 5 × 2 L beakers (0.3 ind mL^−1^). Each beaker had a false bottom which was lined with 150 μm nylon mesh to enable separation of the females from the eggs to prevent egg cannibalism. The beaker was filled with ambient aerated seawater with known saturating prey conditions (1 µg C mL^−1^; prey carbon ratio 1:1:1 of *I. galbana, T. suecica* and *C. muelleri*) and females were left for 5 h during the dark phase to produce eggs. A minimum of 70 eggs were collected for each pCO_2_ treatment level and were utilized for egg hatching rates in the same manner as described above.

### Statistical analysis

The impact of pCO_2_ on the reproductive success of *Acartia tonsa* was analysed using PRIMER-v6. For all variables (EPR, EHS, NP and egg volume), a resemblance matrix was constructed for PERMANOVA analysis, using Euclidean Distance. For each variable, a factorial design with two crossed fixed-factors (experimental design and pCO_2_ concentration) was performed. To enable cross-comparisons between the different experimental designs (MFE, FE and NE), the pCO_2_ concentrations were allocated into levels 1–5 (385, 1000, 2000, 3000 and 6000 µatm, respectively). Main effects and pairwise comparisons of the different factors were analysed through unrestricted permutations of raw data. If a low number of permutations were generated then the *P*-value was obtained through random sampling of the asymptotic permutation distribution, using Monte Carlo tests. For each variable, the dispersion across the factors was first analysed using PERMDISP, which indicated that both EHS and egg volume had a significantly different dispersion across the different pCO_2_ levels (both, *P* = <0.05). To minimize this effect, both EHS and egg volume were transformed (log (χ+ 1)) prior to the PERMANOVA analysis.

## RESULTS

Table I shows the seawater chemistry of the different pCO_2_ treatments across the three different experimental designs. Throughout the following text and figures, reference is made to the nominal (i.e. target) pCO_2_ µatm values, rather than to the precise values given within Table I.

**Table I:** Seawater chemistry parameters for all three experimental designs (mean ± 1 SD)

Experimental design	Physiochemical water properties	Nominal pCO_2_ levels (µatm)				
		385	1000	2000	3000	6000
Male and female exposure (MFE)	Male pH^a^	8.235 (±0.007)	7.818 (±0.004)	7.610 (±0.004)	7.411 (±0.004)	7.149 (±0.007)
	Male pH^b^	8.218 (±0.009)	7.814 (±0.005)	7.608 (±0.007)	7.403 (±0.065)	7.153 (±0.004)
	Female pH^a^	8.235 (±0.007)	7.818 (±0.004)	7.610 (±0.004)	7.411 (±0.004)	7.149 (±0.007)
	Female pH^b^	8.222 (±0.008)	7.817 (±0.005)	7.614 (±0.005)	7.417 (±0.005)	7.151 (±0.005)
	Egg hatching pH^a^	8.235 (±0.007)	7.818 (±0.004)	7.610 (±0.004)	7.411(±0.004)	7.149 (±0.007)
	Egg hatching pH^b^	8.193 (±0.023)	7.832 (±0.011)	7.666 (±0.016)	7.526 (±0.033)	7.295 (±0.050)
	A*_T_* (µmol kg^−1^)	2435 (±59.8)	2336 (±27.15)	2400 (±40.31)	2331 (±54.02)	2404 (±93.20)
	pCO_2_ (µatm)^c^	400 (±10.93)	1142 (±14.95)	1972 (±30.61)	3071 (±59.61)	5924 (±194.15)
	Temperature (°C)	23.87 (±0.15)	23.86 (±0.05)	23.93 (±0.05)	23.90 (±0.05)	23.88 (±0.05)
	Salinity	27.73 (±0.08)	27.7 (±0.09)	27.63 (±0.05)	27.83 (±0.10)	27.73 (±0.05)
Sole female exposure (FE)	Female pH^a^	8.206 (±0.004)	7.867 (±0.005)	7.592 (±0.005)	7.443 (±0.007)	7.147 (±0.007)
	Female pH^b^	8.193 (±0.014)	7.860 (±0.005)	7.602 (±0.007)	7.450 (±0.005)	7.154 (±0.007)
	Egg hatching pH^a^	8.206 (±0.004)	7.867 (±0.005)	7.592 (±0.005)	7.443 (±0.007)	7.147 (±0.007)
	Egg hatching pH^b^	8.185 (±0.019)	7.891 (±0.021)	7.635 (±0.010)	7.470 (±0.010)	7.184 (±0.011)
	A*_T_* (µmol kg^−1^)	2416 (±37.83)	2383 (±44.68)	2360 (±69.29)	2382 (±14.85)	2414 (±62.30)
	pCO_2_ (µatm)^c^	427 (±5.15)	1033 (±14.95)	2022 (±44.65)	2919 (±13.73)	5959 (±116.30)
	Temperature (°C)	24.04 (±0.05)	24.08 (±0.08)	24.20 (±0.02)	24.14 (±0.05)	24.10 (±0.09)
	Salinity	28.01(±0.02)	27.84 (±0.05)	27.94 (±0.05)	28.00 (±0.00)	28.09 (±0.02)
No parental exposure (NE)	pH^a^	8.255 (±0.005)	7.907 (±0.003)	7.614 (±0.007)	7.424 (±0.013)	7.143 (±0.004)
	pH^b^	8.171 (±0.031)	7.926 (±0.125)	7.666 (±0.023)	7.510 (±0.034)	7.313 (±0.038)
	A*_T_* (µmol kg^−1^)	2412 (±54.73)	2401 (±11.91)	2398 (±25.00)	2417 (±19.9)	2349 (±70.9)
	pCO_2_ (µatm)^c^	375 (±8.36)	940 (±8.70)	1946 (±34.00)	3092 (±94.10)	5875 (±143.70)
	Temperature (°C)	24.41 (±0.03)	24.13 (±0.19)	24.26 (±0.15)	24.13 (±0.05)	24.36 (±0.05)
	Salinity	27.86 (±0.07)	27.86 (±0.05)	28.05 (±0.05)	27.86 (±0.05)	28.08 (±0.05)

^a^Average initial pH concentrations.

^b^Average pH concentrations before the 95% water exchange (which occurred every 24 h with FE and MFE adults, and after 96 h for eggs across all three experiments). All pH values are against the NBS scale.

^c^Parameters calculated through CO_2_ SYS (Pierrot *et al.*, 2006).

**Fig. 2.**
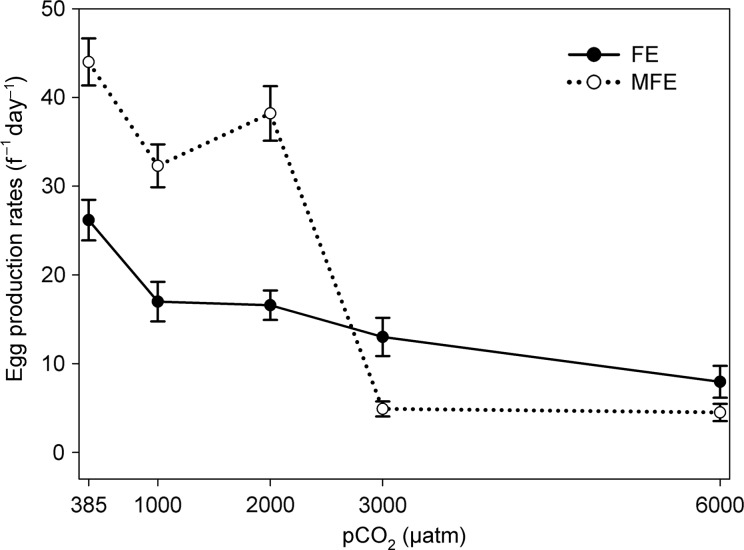
Egg production rates (mean ± 1 SE) of *Acartia tonsa* exposed to five different pCO_2_ levels with different parental exposures: MFE, combined male and female exposure to the pCO_2_ level; FE, sole female exposure to the pCO_2_ level; NE, no parental exposure to the pCO_2_ level.

EPR declined with increasing pCO_2_ exposure within both experimental designs; MFE: *F* = 73.69, *P* = 0.001, and FE: *F* = 9.64, *P* = 0.001, (Fig. 2). The EPR differed significantly between the control groups of MFE and FE (*t* = 20.27, *P* = 0.001), highlighting the different reproductive outcome between the experimental designs under existing ambient conditions (Fig. 2). For this reason, all EPR were first normalized against their individual controls before comparing the normalized EPR (EPR^norm^) between the different experimental designs within each pCO_2_ level. Significant differences were found between the EPR^norm^, with significantly lower rates with MFE compared with FE at 3000 µatm (*t* = 4.02 *P* = 0.001) and 6000 µatm pCO_2_ *(t* = 2.68, *P* = 0.008). However, no difference was found in the EPR^norm^ of the females for the projected 2100 CO_2_ scenarios (1000 µatm CO_2_, Fig. 2) between MFE and FE.

**Fig. 3.**
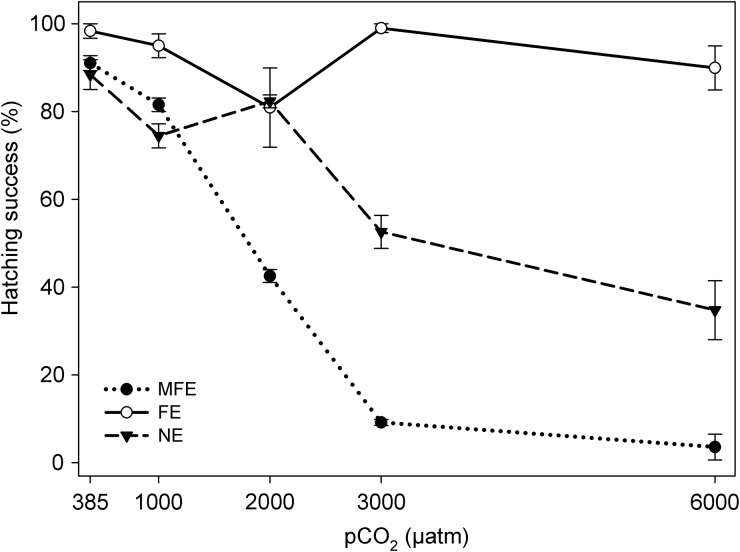
Egg hatching success (mean ± 1 SE) of *Acartia tonsa* exposed to five different pCO_2_ levels with different parental exposures: MFE, combined male and female exposure to the pCO_2_ level; FE, sole female exposure to the pCO_2_ level; NE, no parental exposure to the pCO_2_ level.

Using the FE protocol, no correlation was found between egg hatching success (EHS) and pCO_2_ concentration, highlighting offspring resilience to pCO_2_ levels up to 6000 µatm when adopting this experimental design (Fig. 3). In stark contrast, using the MFE protocol, EHS significantly declined with every successive pCO_2_ concentration. Upon exposure to the 2100 year scenario (1000 µatm pCO_2_), eggs produced with the MFE protocol showed a significant decline in hatching success (*t* = 4.15, *P* = 0.014) by 10%. This decline increased to >45% at 2000 µatm CO_2_ (*t* = 19.60, *P* = 0.001), and to >90% upon exposure to 3000 and 6000 µatm pCO_2_ (both, *P* < 0.01). Similar to MFE, declines in EHS with increased pCO_2_ concentration were found in eggs with NE (*F* = 20.42, *P* = 0.001), but this decline was only significant in the two highest pCO_2_ concentrations (3000 µatm, *t* = 6.69, *P* = 0.024; 6000 µatm, *t* = 5.19, *P* = 0.036). Hatching success significantly varied between all three experimental approaches (*F* = 118.90*, P* = 0.001). This variation correlated with an increase in pCO_2_ concentrations (*F* = 33.25*, P* = 0.001). The greatest variation found was between FE and MFE (*t* = 4.49*, P* = 0.001), followed by FE and NE (*t* = 4.33*, P* = 0.001), and with the least variation found between NE and FE (*t* = 2.80*, P* = 0.004).

**Fig. 4.**
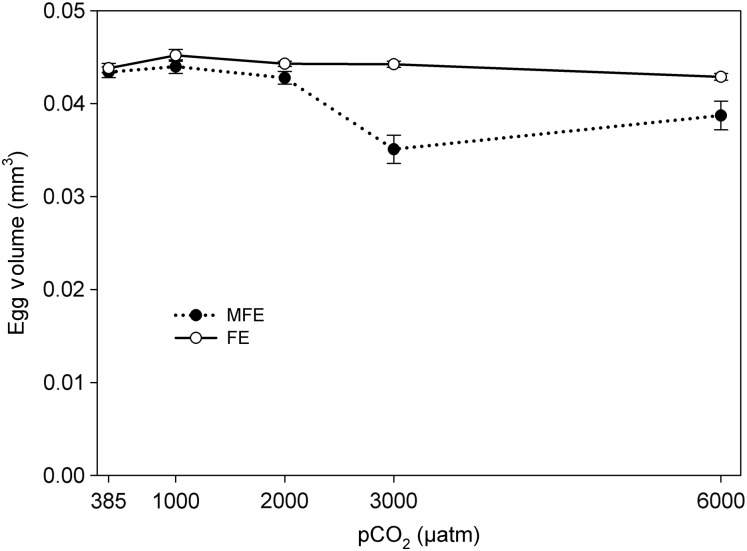
Egg volume (mean ± 1 SE) of *Acartia tonsa* exposed to five different pCO_2_ levels with different parental exposures: MFE, combined male and female exposure to the pCO_2_ level;, FE, sole female exposure to the pCO_2_ level.

Egg volume significantly differed between FE and MFE (*F* = 50.19, *P* = 0.001); declines in egg volume were found with elevated pCO_2_ concentrations in MFE (3000 µatm, *t* = 5.76*, P* = 0.001; 6000 µatm, *t* = 3.31*, P* = 0.002). In contrast, the volumes of the eggs produced by FE were not affected at any pCO_2_ concentration (Fig. 4). As such, the variation in egg volumes between FE and MFE significantly differed at 2000 µatm (*t* = 2.31*, P* = 0.03), 3000 µatm (*t* = 6.38*, P* = 0.001) and 6000 µatm (*t* = 3.03*, P* = 0.002).

**Fig. 5.**
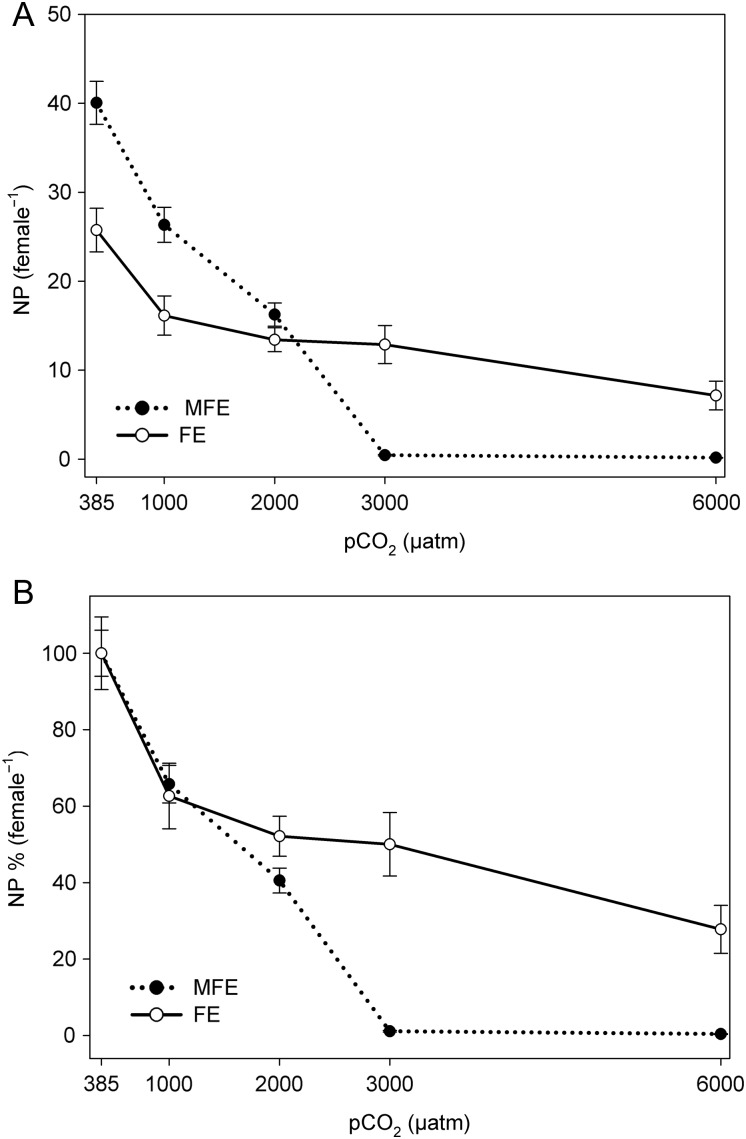
Nauplii production (mean ± 1 SE) of *Acartia tonsa* exposed to five different pCO_2_ levels with different parental exposures: (**A**) nauplii production (mean ± 1 SE) and (**B**) production normalized (i.e. NP^norm^) against individual treatment controls at extant pCO_2_. MFE, combined male and female exposure to the pCO_2_ level; FE, sole female exposure to the pCO_2_ level.

Combining EPR and EHS (i.e. EPR × EHS) revealed a decline in the NP with elevated pCO_2_ in both MFE (*F* = 128.69*, P* = 0.001) and FE (*F* = 11.15*, P* = 0.001). In both MFE and FE, the NP significantly declined with each successive pCO_2_ concentration (Fig. 5A), but this rate of decline was greater in MFE than FE at ≥2000 µatm. Similar to the EPR, the NP significantly differed between the control groups (Fig. 5A); Fig. 5B shows these data again, but normalized against their individual controls (i.e. ambient pCO_2_; NP^norm^). Variation in NP^norm^ between the different experiments differed significantly at 3000 µatm (*t* = 5.146*, P* = 0.001) and 6000 µatm pCO_2_ (*t* = 4.153*, P* = 0.001).

## DISCUSSION

While the effect of elevated pCO_2_ on female copepod fecundity has been the subject of significant research activity, there is very limited information available on the effects of elevated pCO_2_ on copepod reproductive success with combined MFE; as highlighted in Supplementary data, Appendix S1. Parental pre-exposure to high pCO_2_ has previously been shown to influence the outcome of the future progeny across marine species (Miller *et al*, 2012; Parker *et al,* 2012; Allen *et al,* 2014). Within this present study, we have found substantial variations in the reproductive success of the calanoid copepod, *Acartia tonsa*, dependent on whether the parents were pre-exposed to the pCO_2_ concentration, and indeed, whether both parents were pre-exposed or just the females (Figs 2–5).

### Egg production

EPR declined significantly with elevated pCO_2_ under both combined MFE and sole FE protocols. This decline in EPR occurred at much lower pCO_2_ than that seen in other species (see summarized Table in Supplementary data, Appendix S1), which may indicate particular sensitivity of *Acartia tonsa* to increased pCO_2_. At extreme pCO_2_ concentrations (≥3000 µatm), beyond the climate change projection scenarios, the EPR from the MFE protocol was significantly lower than that obtained using the FE protocol (Fig. 2). Previously, declines in EPR have been attributed to the suppression in metabolic activity through decreased protein synthesis, consequently decreasing reproductive output (Kurihara, 2008). Within *Acartia*, all developmental oocyte stages are present at any one time within the gonads of the female which enables the maturation of the oocytes to be a continuous process, facilitating the frequent spawning events by this species (Niehoff, 2007). If the extreme elevated pCO_2_ levels (≥3000 µatm) were to affect female oocyte development and result in decreased egg production, it would be seen in the females across both experimental designs (i.e. MFE and FE), occurring at a similar rate of decline. However, this was not the case; the rate of decline in egg production was much greater using the MFE protocol than FE (Fig. 2). This is suggestive of an additive effect on EPR, either as a result of the paternal exposure to the pCO_2_, or a combined effect of maternal and paternal exposure.

The paternal influence on EPR is likely to be due to the effects of pCO_2_ on spermatogenesis within the exposed males, as females in the FE protocol, with a pre-attached spermatophore produced by males under ambient conditions, were less affected at high pCO_2_ concentrations (Fig. 2). The effect of elevated pCO_2_ may interact with spermatogenesis within males affecting production and development of the spermatozoa, seminal fluids or even the structure of the spermatophore itself (Fitzer *et al.,* 2012a). Although reproductive behaviour post-copulation has scarcely been researched in copepods, it has been suggested that females have the ability to detach unwanted spermatophores and display post-copulatory mate choice (Titelman *et al.*, 2007). This could explain the cessation of egg production seen with the MFE protocol at ≥3000 µatm pCO_2_.

### Egg hatching success

By far the greatest differences between parental exposures were seen in the EHS rates (Fig. 3). Variations between the experimental protocols positively correlated (*R* = 0.94) with an increase in pCO_2_ concentrations; with more than 20% variation in EHS found in the 2100 year pCO_2_ scenarios (1000 µatm), increasing to >85% at 6000 µatm pCO_2_. The elevated pCO_2_ led to a decreased hatching success of eggs at every test concentration with MFE, yet no effect was found on the EHS at any pCO_2_ concentration with FE. This indicates that a substantial proportion of eggs produced through MFE were either: (i) unfertilized oocytes, (ii) non-viable fecund eggs, or (iii) viable fecund eggs in a quiescent state.

### Unfertilized oocytes

The production of unfertilized oocytes under MFE may result from either female sterile egg production or a limitation in fertilization success as a result of male exposure to pCO_2_ (or indeed a combined synergistic MFE effect). Previously, production of sterile eggs in copepods has been associated with limiting male abundance and hence the decreasing scope for the females to re-mate leading to declining numbers of spermatophores available for reproduction (Parrish and Wilson, 1978; Titelman *et al.*, 2007). As each female used for egg production and hatching success in our experiments had an attached spermatophore, this explanation is unlikely to be acceptable. Alternatively, unfertilized oocytes could have been produced from the female as a result of male gamete imperfections associated with the added paternal exposure to the high pCO_2_. If this was so, then the effect of high pCO_2_ on the male gamete is likely to have occurred during the process of spermatogenesis (refer to Fig. 1A), as eggs produced by FE (using females that had a pre-attached spermatophore produced from males under extant conditions) maintained a high hatching success rate that was not influenced by elevated pCO_2_ (Fig. 3).

In copepods, the aflagellate and immobile spermatozoa are transferred in spermatophores to the females. The production of these spermatophores, and their contents, occurs entirely within the male reproductive system (e.g. *Acartia*, Fig. 1A). The discharge of the spermatozoa from the spermatophore occurs through the hydrostatic and mechanical pressure associated with the uptake of water from the inner cell walls of spermatophore (Blades-Eckelbarger, 1991). As far as is known only one study (Fitzer *et al.*, 2012a) has measured the impacts of OA on male copepod gametes (*Tisbe battaglia*), which found that the spermatophores attached to the females reared under pH 7.67 showed a decreased chitinous structural appearance in the spermatophore wall, compared with those reared under ambient conditions (pH 8.01). If the degree of deterioration seen in the spermatophore walls is such that it impacts on the cells within the cell wall [which aid the ejection and discharge of the spermatozoa from the spermatophore (Blades-Eckelbarger, 1991)] then the reproductive success of the female would be hindered by the quantity of the spermatozoa which are actually able to be ejected for fertilization. This would significantly affect the quantity of eggs that would be fertilized upon release (Fig. 1A), which could aid in explaining the decline in EHS (Fig. 3) and steeper decline in NP (Fig. 5) seen with MFE in comparison with FE.

Prior OA studies have found the paternal influence in marine invertebrates to be a potential limiting factor in reproduction, with declines in sperm numbers (Reuter *et al.,* 2011) and motility (Havenhand *et al.,* 2008; Morita *et al.*, 2010; Vihtakari *et al.,* 2013) influencing offspring success. Fitzer *et al*. (Fitzer *et al.*, 2012a) found significant declines in spermatophore length in *Tisbe battaglia* with increased acidity, compared with that of ambient conditions. This alludes to the potential decease in the seminal products available within the spermatophore to fertilize the mature eggs, resulting in an increased count of unfertilized oocytes. However, spermatophore length is not necessarily proportional to the abundance of spermatozoa, and the potential for the spermatophore to contain other substances has been suggested (Sichlau and Kiorboe, 2011). These elusive substances may be vital for reproductive success and aid in the spermatophore being accepted by the female, e.g. potential nuptial gift for the mother to enhance reproductive success [hormones, proteins, lipids (Titelman *et al.*, 2007)]. Thus the effect of high pCO_2_ on the spermatophore may not only influence the spermatozoa within, but also other substances vital for reproductive success, impacting on fertilization success, egg viability and quality. These impacts occurring on the male gametes through spermatogenesis could also account for the declines in EHS and NP found with elevated pCO_2_ with MFE and not found with FE.

### Non-viable fecund eggs

The eggs produced with no prior exposure to pCO_2_ (NE protocol) were assumed to be fertilized (i.e. not sterile) under ambient conditions prior to being exposed to the different pCO_2_ levels; in support of this, the proportion hatching under ambient conditions was >95% (Fig. 3). Thus the decline in hatching success of the eggs exposed to >2000 µatm pCO_2_ is either a consequence of non-viable fecund eggs or fecund eggs in a resting state. Non-viable fecund eggs would have resulted from adverse effects during the developmental stages of embryogenesis; e.g. cleavage, blastulation, gastrulation and organogenesis, which would have prevented the formation of the zygote (e.g. Fig. 1C). Equally if the eggs produced within the MFE protocol were fertilized, rather than being unfertilized oocytes, then the decline in hatching success with increased pCO_2_ (Fig. 3) could similarly be attributed to abnormal embryonic development. A previous study has suggested that the declines in the reproductive success of *Calanus finmarchicus* exposed to 8000 ppm CO_2_ could be attributed to adverse effects on the acrosome reaction, or as a result of polyspermy (Mayor *et al.,* 2007). If so, this could present a route through which high pCO_2_ may influence the first and/or second binding of the two haploid gametes affecting their ability to fuse and form a diploid, resulting in the declined hatching success of the eggs produced.

### Fecund eggs in a resting state

An alternate explanation would see the declined hatching success under the MFE protocol, and to a lesser extent under the NE protocol, as a result of viable fecund eggs being held in a resting state. The arrestment of embryogenesis development is a physiological response to adverse environmental conditions that enables subitaneous eggs to enter a quiescent state until conditions become more favourable. Alterations in environmental conditions, such as low temperature (Drillet *et al*., 2006) anoxia and abrupt changes in salinity (Holmstrup *et al*., 2006), have been shown to induce quiescence in *Acartia* eggs. Indeed, internal pH has shown to regulate diapause in embryos, with external fluctuations in pH being a factor influencing the resting state of the embryo in *Artemia* (Sedlacek, 2008). However, if increased external pCO_2_ were to influence the resting state of *Acartia* eggs with MFE and indeed NE, it would also be expected in eggs with FE, but this was not the case. As such, the liberation of resting stage eggs is not likely to be a factor found within our set of experiments.

The sole exposure of females to increased pCO_2_ (FE protocol) has demonstrated the resilience in the hatching success of the future progeny, compared with those unfertilized oocytes or non-viable fecund eggs that were produced with combined MFE. This variability highlights the opposing reproductive outcomes dependent on parental exposure. Elevated pCO_2_ resulted in decreased egg production under the FE protocol and almost cessation in the MFE protocol. However, those eggs produced under the FE protocol hatched 80–98% irrespective of the external pCO_2_ levels (Fig. 3), resulting in a greater production of nauplii compared with MFE (Fig. 5B). Furthermore, the size of the eggs produced through FE was not affected by elevated pCO_2_ at any test concentration, but the eggs produced by MFE decreased significantly in volume at the higher pCO_2_ levels (Fig. 4). Perhaps, the increased resilience of the offspring survival to elevated pCO_2_ seen with sole FE, compared with the additive paternal influence seen with MFE, illustrates a maternal response to the short-term elevations in pCO_2_, i.e. AMEs; fewer progeny but of better quality and ability to hatch. If this is a response to a rapid change in carbonate chemistry over a critical period of time, this result from FE may not mimic what will be seen with OA in the wild.

## CONCLUSION

In comparison with females, little is known of the male reproductive biology in copepods, limiting understanding of any associated paternal cause-and-effect of high pCO_2_ on reproductive success. However, with prior paternal limitations found in other marine organisms under high pCO_2_ (Caldwell *et al.*, 2011), along with the variation in reproductive success seen between the different experimental designs within this present study, there is a clear need for further research in this area to prevent misrepresentation and error propagation. The vast majority of copepod reproductive measurements have been carried out solely on wild caught females, as seen in Supplementary data, Appendix S1. Indeed, this over-reliance on results from female copepods is common across copepod research (Mitra *et al*., 2014), notably with OA (Cripps *et al.*, 2014). In stark contrast to the results using the FE protocol, the lower numbers of eggs produced by MFE exposed to elevated pCO_2_ were coupled with a decline in hatching success (Fig. 3). This suggests a paternal limitation in reproductive success, or a combined maternal and paternal effect. The final result (Fig. 5) is one that does not appear to bode well for copepods under OA in the near-future.

## SUPPLEMENTARY DATA

Supplementary data can be found online at http://plankt.oxfordjournals.org.

## FUNDING

This project was funded by the Natural Environmental Research Council PhD case studentship between Plymouth Marine Laboratory and Swansea University, and by Natural Environment Reasearch Council UK grant NE/H01750X/1 to KJF. Funding to pay the Open Access publication charges for this article was provided by Research Councils UK.

## Supplementary Material

Supplementary Data
