# Supplementary material for: Parental exposure to elevated pCO2 influences the reproductive success of copepods
Source: J Plankton Res. 2014 Jun 19;36(5):1165–74. doi: 10.1093/plankt/fbu052 (PMC4161228; doi:10.1093/plankt/fbu052)
Supplement: Supplementary Data [file supp_36_5_1165__index.html]

Parental exposure to elevated pCO2 influences the reproductive success of copepods — Supplementary Data 

# Parental exposure to elevated pCO2 influences the reproductive success of copepods

## Supplementary Data

Supplementary Data

**Files in this Data Supplement:**

- Supplementary Data - Docx file
