# Supplementary material for: Parental exposure to elevated pCO2 influences the reproductive success of copepods
Source: J Plankton Res. 2014 Jun 19;36(5):1165–74. doi: 10.1093/plankt/fbu052 (PMC4161228; doi:10.1093/plankt/fbu052)
Supplement: Supplementary Data [file supp_fbu052_fbu052supp.docx]

**Appendix** (Supplementary Material online)**:**

**Appendix 1:** The reproductive response of different copepod species exposed to high pCO_2_/ low pH, categorised into different parental exposures; FE = studies which measured the reproductive success through sole female exposure to the high pCO_2_/ low pH (see Fig. 1B). MFE = studies which measured the reproductive success through combined male and female exposure to the high pCO_2_/ low pH (Fig. 1A). The horizontal dotted line marks the cut off of the 2100 pCO_2_/ pH scenarios; below this line are listed studies using scenarios beyond climate change projections. Reproductive responses: ↔ = no effect, ↓ = negative effect, ↑ = positive effect. EPR = egg production rate, EHR = egg hatching rate, NP = nauplii production.

| **Species** | **FE ( ♀) MFE ( ♀♂)** |  | **Duration** | **pH/CO_2_** |  | **Response** | | **Reference** |
| --- | --- | --- | --- | --- | --- | --- | --- | --- |
|  |  |  |  |  |  |  |  |  |
| *Acartia clausi* | ♀ |  | 1-2 d | pH 7.83 |  | **↔** | EPR | Zervoudaki *et al,* 2013 |
| *Acartia pacifica* | ♀ |  | 8 d | pH 7.84-7.85 |  | **↔** | EPR | Zhang *et al*, 2011 |
| *Acartia spinicauda* | ♀ |  | 8 d | pH 7.84-7.85 |  | **↔** | EPR | Zervoudaki *et al,* 2013 |
| ***Acartia tonsa*** | ♀♂ |  | 4 d | 1000 µatm |  | **↓** | EPR | Present study |
| ***Acartia tonsa*** | ♀ |  | 4 d | 1000 µatm |  | **↓** | EPR | Present study |
| *Acartia tonsa* | ♀♂ |  | 12 d | 750 µatm |  | **↓** | EPR | Rossoll *et al,* 2012 |
| *Calanus sinicus* | ♀ |  | 8 d | pH 7.84-7.85 |  | **↔** | EPR | Zervoudaki *et al,* 2013 |
| *Centropages tenuiremis* | ♀ |  | 8 d | pH 7.84-7.85 |  | **↔** | EPR | Zhang *et al*, 2011 |
| *Centropages typicus* | ♀ |  | 4 d | 480 ppm |  | **↔** | EPR | McConville *et al*, 2012 |
| *Centropages typicus* | ♀ |  | 4 d | 620 ppm |  | **↔** | EPR | McConville *et al*, 2012 |
| *Centropages typicus* | ♀ |  | 4 d | 750 ppm |  | **↔** | EPR | McConville *et al*, 2012 |
| *Temora longicornis* | ♀ |  | 4 d | 480 ppm |  | **↔** | EPR | McConville *et al*, 2012 |
| *Temora longicornis* | ♀ |  | 4 d | 620 ppm |  | **↔** | EPR | McConville *et al*, 2012 |
| *Temora longicornis* | ♀ |  | 4 d | 750 ppm |  | **↔** | EPR | McConville *et al*, 2012 |
|  |  |  |  |  |  |  |  |  |
| *Acartia clausi* | ♀ |  | 1-2 d | pH 7.83 |  | **↔** | EHR | Zervoudaki *et al,* 2013 |
| ***Acartia tonsa*** | ♀♂ |  | 4 d | 1000 µatm |  | **↓** | EHR | Present study |
| ***Acartia tonsa*** | ♀ |  | 4 d | 1000 µatm |  | **↔** | EHR | Present study |
| *Centropages typicus* | ♀ |  | 4 d | 480 ppm |  | **↔** | EHR | McConville *et al*, 2012 |
| *Centropages typicus* | ♀ |  | 4 d | 620 ppm |  | **↔** | EHR | McConville *et al*, 2012 |
| *Centropages typicus* | ♀ |  | 4 d | 750 ppm |  | **↔** | EHR | McConville *et al*, 2012 |
| *Temora longicornis* | ♀ |  | 4 d | 480 ppm |  | **↔** | EHR | McConville *et al*, 2012 |
| *Temora longicornis* | ♀ |  | 4 d | 620 ppm |  | **↔** | EHR | McConville *et al*, 2012 |
| *Temora longicornis* | ♀ |  | 4 d | 750 ppm |  | **↔** | EHR | McConville *et al*, 2012 |
|  |  |  |  |  |  |  |  |  |
| ***Acartia tonsa*** | ♀♂ |  | 4 d | 1000 µatm |  | **↓** | NP | Present study |
| ***Acartia tonsa*** | ♀ |  | 4 d | 1000 µatm |  | **↓** | NP | Present study |
| *Tisbe battagliai* | ♀♂ |  | 12 d | pH 7.94 |  | **↑** | NP | Fitzer *et al*, 2012 (a) |
| *Tisbe battagliai* | ♀♂ |  | 24 d | pH 7.94 |  | **↓** | NP | Fitzer *et al*, 2012 (a) |
| *Tisbe battagliai* | ♀♂ |  | 36 d | pH 7.94 |  | **↓** | NP | Fitzer *et al*, 2012 (a) |
| *Tisbe battagliai* | ♀♂ |  | 12 d | pH 7.82 |  | **↓** | NP | Fitzer *et al*, 2012 (a) |
| *Tisbe battagliai* | ♀♂ |  | 24 d | pH 7.82 |  | **↔** | NP | Fitzer *et al*, 2012 (a) |
| *Tisbe battagliai* | ♀♂ |  | 36 d | pH 7.82 |  | **↔** | NP | Fitzer *et al*, 2012 (a) |
| *Tisbe battagliai* | ♀♂ |  | 12 d | pH 7.95 |  | **↔** | NP | Fitzer *et al,* 2013 |
| *Tisbe battagliai* | ♀♂ |  | 24 d | pH 7.95 |  | **↓** | NP | Fitzer *et al,* 2013 |
| *Tisbe battagliai* | ♀♂ |  | 12 d | pH 7.82 |  | **↓** | NP | Fitzer *et al,* 2013 |
| *Tisbe battagliai* | ♀♂ |  | 24 d | pH 7.82 |  | **↔** | NP | Fitzer *et al,* 2013 |
|  |  |  |  |  |  |  |  |  |
|  |  |  |  |  |  |  |  |  |
| *Acartia steueri* | ♀ |  | 8 d | pH 7.40-7.55 |  | **↔** | EPR | Kurihara *et al*, 2004 (a) |
| *Acartia steueri* | ♀ |  | 8 d | pH 6.84-7.02 |  | **↓** | EPR | Kurihara *et al,* 2004 (a) |
| *Acartia erythraea* | ♀ |  | 8 d | pH 7.02-7.08 |  | **↔** | EPR | Kurihara *et al*, 2004 (b) |
| *Acartia erythraea* | ♀ |  | 8 d | pH 6.86-6.96 |  | **↓** | EPR | Kurihara *et al,* 2004 (a) |
| *Acartia pacifica* | ♀ |  | 8 d | pH 7.39-7.37 |  | **↔** | EPR | Zhang *et al*, 2011 |
| *Acartia pacifica* | ♀ |  | 8 d | pH 7.19-7.24 |  | **↔** | EPR | Zhang *et al,* 2011 |
| *Acartia spinicauda* | ♀ |  | 8 d | pH 7.39-7.37 |  | **↔** | EPR | Zhang *et al*, 2011 |
| *Acartia spinicauda* | ♀ |  | 8 d | pH 7.19-7.24 |  | **↓** | EPR | Zhang *et al,* 2011 |
| *Acartia spinicauda* | ♀ |  | 8 d | pH 6.92-6.94 |  | **↓** | EPR | Zhang *et al,* 2011 |
| ***Acartia tonsa*** | ♀♂ |  | 96 h | 2000 µatm |  | **↔** | EPR | Present study |
| ***Acartia tonsa*** | ♀ |  | 96 h | 2000 µatm |  | **↓** | EPR | Present study |
| ***Acartia tonsa*** | ♀♂ |  | 96 h | 3000 µatm |  | **↓** | EPR | Present study |
| ***Acartia tonsa*** | ♀ |  | 96 h | 3000 µatm |  | **↓** | EPR | Present study |
| ***Acartia tonsa*** | ♀♂ |  | 96 h | 6000 µatm |  | **↓** | EPR | Present study |
| ***Acartia tonsa*** | ♀ |  | 96 h | 6000 µatm |  | **↓** | EPR | Present study |
| *Calanus finmarchicus* | ♀ |  | 5 d | 8000 ppm |  | **↔** | EPR | Mayor *et al,* 2007 |
| *Calanus glacialis* | ♀ |  | 7 d | pH 7.6 |  | **↔** | EPR | Weydmann *et al,* 2012 |
| *Calanus glacialis* | ♀ |  | 7 d | pH 6.9 |  | **↔** | EPR | Weydmann *et al*, 2012 |
| *Calanus sinicus* | ♀ |  | 8 d | pH 7.39-7.37 |  | **↔** | EPR | Zhang *et al*, 2011 |
| *Calanus sinicus* | ♀ |  | 8 d | pH 7.19-7.24 |  | **↔** | EPR | Zhang *et al,* 2011 |
| *Calanus sinicus* | ♀ |  | 8 d | pH 6.92-6.94 |  | **↓** | EPR | Zhang *et al,* 2011 |
| *Centropages tenuiremis* | ♀ |  | 8 d | pH 7.39-7.37 |  | **↓** | EPR | Zhang *et al*, 2011 |
| *Centropages tenuiremis* | ♀ |  | 8 d | pH 7.19-7.24 |  | **↓** | EPR | Zhang *et al,* 2011 |
| *Centropages tenuiremis* | ♀ |  | 8 d | pH 6.92-6.94 |  | **↓** | EPR | Zhang *et al,* 2011 |
| *Centropages typicus* | ♀ |  | 4 d | 9830 ppm |  | **↔** | EPR | McConville *et al*, 2012 |
| *Temora longicornis* | ♀ |  | 4 d | 9830 ppm |  | **↓** | EPR | McConville *et al*, 2012 |
|  |  |  |  |  |  |  |  |  |
| *Acartia erythraea* | ♀ |  | 8 d | pH 7.02-7.08 |  | **↔** | EHR | Kurihara *et al*, 2004 (b) |
| *Acartia erythraea* | ♀ |  | 8 d | pH 6.86-6.96 |  | **↓** | EHR | Kurihara *et al,* 2004 (a) |
| *Acartia spinicauda* | ♀ |  | 8 d | pH 7.39-7.37 |  | **↓** | EHR | Zhang *et al,* 2011 |
| *Acartia spinicauda* | ♀ |  | 8 d | pH 6.92-6.94 |  | **↓** | EHR | Zhang *et al,* 2011 |
| ***Acartia tonsa*** | ♀♂ |  | 96 h | 2000 µatm |  | **↓** | EHR | Present study |
| ***Acartia tonsa*** | ♀ |  | 96 h | 2000 µatm |  | **↔** | EHR | Present study |
| ***Acartia tonsa*** | ♀♂ |  | 96 h | 3000 µatm |  | **↓** | EHR | Present study |
| ***Acartia tonsa*** | ♀ |  | 96 h | 3000 µatm |  | **↔** | EHR | Present study |
| ***Acartia tonsa*** | ♀♂ |  | 96 h | 6000 µatm |  | **↓** | EHR | Present study |
| ***Acartia tonsa*** | ♀ |  | 96 h | 6000 µatm |  | **↔** | EHR | Present study |
| *Acartia tonsa* | ♀ |  | 7 d | pH 6.5 |  | **↓** | EHR | Invifia *et al,* 2004 |
| *Calanus finmarchicus* | ♀ |  | 5 d | 8000 ppm |  | **↓** | EHR | Mayor *et al,* 2007 |
| *Calanus glacialis* | ♀ |  | 9d | pH 7.6 |  | **↔** | EHR | Weydmann *et al*, 2012 |
| *Calanus glacialis* | ♀ |  | 9 d | pH 6.9 |  | **↓** | EHR | Weydmann *et al*, 2012 |
| *Centropages tenuiremis* | ♀ |  | 8 d | pH 7.39-7.37 |  | **↓** | EHR | Zhang *et al,* 2011 |
| *Centropages tenuiremis* | ♀ |  | 8 d | pH 6.92-6.94 |  | **↓** | EHR | Zhang *et al,* 2011 |
| *Centropages typicus* | ♀ |  | 4 d | 9830 ppm |  | **↔** | EHR | McConville *et al*, 2012 |
| *Temora longicornis* | ♀ |  | 4 d | 9830 ppm |  | **↓** | EHR | McConville *et al*, 2012 |
|  |  |  |  |  |  |  |  |  |
| ***Acartia tonsa*** | ♀♂ |  | 96 h | 2000 µatm |  | **↓** | NP | Present study |
| ***Acartia tonsa*** | ♀ |  | 96 h | 2000 µatm |  | **↓** | NP | Present study |
| ***Acartia tonsa*** | ♀♂ |  | 96 h | 3000 µatm |  | **↓** | NP | Present study |
| ***Acartia tonsa*** | ♀ |  | 96 h | 3000 µatm |  | **↓** | NP | Present study |
| ***Acartia tonsa*** | ♀♂ |  | 96 h | 6000 µatm |  | **↓** | NP | Present study |
| ***Acartia tonsa*** | ♀ |  | 96 h | 6000 µatm |  | **↓** | NP | Present study |
| *Tisbe battagliai* | ♀♂ |  | 12 d | pH 7.67 |  | **↓** | NP | Fitzer *et al*, 2012 (a) |
| *Tisbe battagliai* | ♀♂ |  | 24 d | pH 7.67 |  | **↔** | NP | Fitzer *et al*, 2012 (a) |
| *Tisbe battagliai* | ♀♂ |  | 36 d | pH 7.67 |  | **↔** | NP | Fitzer *et al*, 2012 (a) |
| *Tisbe battagliai* | ♀♂ |  | 12 d | pH 7.67 |  | **↓** | NP | Fitzer *et al*, 2013 |
| *Tisbe battagliai* | ♀♂ |  | 24 d | pH 7.67 |  | **↔** | NP | Fitzer *et al*, 2013 |

Invidia, M., Sei, S., Gorbi, G. (2004). Survival of the copepod *Acartia tonsa* following egg exposure to near anoxia and to sulphide at different pH levels. *Mar.Ecol.Prog.Ser.* **276**, 187-196.

Kurihara, H., Shimode, S., Shirayama, Y. (2004a) Effects of raised CO_2_ concentration on the egg production rate and early development of two marine copepods (*Acartia steueri* and *Acartia erythraea*). *Mar.Pollut.Bull.*  **49**, 721–727.

Kurihara, H., Shimode, S., Shirayama, Y. (2004b) Sub-lethal effects of elevated concentration of CO_2_ on planktonic copepods and sea urchins. *J.Oceanogr.* **60**, 743–750.

Weydmann, A., Søreide, J.E., Kwasniewski, S. *et al* (2012) Influence of CO_2_ -induced acidification on the reproduction of a key Arctic copepod *Calanus glacialis.* *J.Exp.Mar.Biol.Ecol.* **428**, 39–42.

Zervoudaki, S., Christou, E.D., Assimakopoulou. G. *et al* (2011) Copepod communities, production and grazing in the Turkish Straits System and the adjacent northern Aegean Sea during spring. *J.Marine.Sys*. **86**, 45–56.

Zhang, D., Li, S., Wang, G. *et al* (2011) Impacts of CO_2_-driven seawater acidification on survival, egg production rate and hatching success of four marine copepods. *Acta*.*Oceanologica Sinica*. **30**, 86–94.
